# Astrocyte-Derived Extracellular Vesicles for Ischemic Stroke: Therapeutic Potential and Prospective

**DOI:** 10.14336/AD.2023.0823-1

**Published:** 2024-05-07

**Authors:** Xianghui Wang, Aihua Li, Huaju Fan, Yanyan Li, Nana Yang, Yaohui Tang

**Affiliations:** ^1^School of Bioscience and Technology, Weifang Medical University, Weifang, Shandong, China.; ^2^School of Biomedical Engineering and Affiliated Sixth People’s Hospital, Shanghai Jiao Tong University, Shanghai, China.; ^3^Department of rehabilitation medicine, Jinan Hospital, Jinan, China

**Keywords:** astrocytes, extracellular vesicles, ischemic stroke

## Abstract

Stroke is a leading cause of death and disability in the world. Astrocytes are special glial cells within the central nervous system and play important roles in mediating neuroprotection and repair processes during stroke. Extracellular vesicles (EVs) are lipid bilayer particles released from cells that facilitate intercellular communication in stroke by delivering proteins, lipids, and RNA to target cells. Recently, accumulating evidence suggested that astrocyte-derived EVs (ADEVs) are actively involved in mediating numerous biological processes including neuroprotection and neurorepair in stroke and they are realized as an excellent therapeutic approach for treating stroke. In this review we systematically summarize the up-to-date research on ADEVs in stroke, and prospects for its potential as a novel therapeutic target for stroke. We also provide an overview of the effects and functions of ADEVs on stroke recovery, which may lead to developing clinically relevant therapies for stroke.

## 1. Introduction

### 1.1 Brief overview of ischemic stroke

Stroke is a devastating disease caused by acute local injury of vascular in the brain [1]. According to statistics, stroke accounts for the second largest cause of death in the world [2], and 17% of people will experience stroke in his life, and 10% of them will die because of stroke [3, 4]. Stroke is mainly divided into two main types: ischemic stroke and hemorrhagic stroke [5]. Ischemic stroke, caused by cerebral arterial occlusion, is responsible for the majority of stroke [6], which is featured by acute dysfunction of neurons, astroglia, oligodendroglia and synaptic architecture [7]. Currently, the main treatments for ischemic stroke depend on restoring oxygen and glucose supply to the ischemic area via drugs or early thrombolysis [8, 9]. However, recombinant t-PA (rt-PA) has some limitations, such as a short treatment window, an extremely short half-life, and limited penetration through large clots [10, 11].

### 1.2 Significance of astrocytes in the pathophysiology and recovery of ischemic stroke

After the occurrence of ischemic stroke, a large number of astrocytes activate and play an important regulatory role [12]. Ischemic stroke can trigger inflammatory response. Astrocytes recruit immune cells to reach the damaged area and participate in inflammatory response regulation and repair by producing a series of cytokines and chemokines [13]. In addition, astrocytes can release various growth factors and metabolites to regulate synaptic activity and promote the survival and regeneration of neurons [14]. As an important part of the blood-brain barrier (BBB), astrocytes form a capsule around the blood vessels, covering a large area of the surface of cerebral blood vessels and regulating cerebral blood flow, which can participate in the repair and reconstruction of the BBB after stroke [15]. Astrocytes can establish connections with many cells, including neurons and their synapses, oligodendrocyte progenitor cells (OPC), oligodendrocytes, microglia, meningeal fibroblasts, circulating immune cells, and various perivascular cells via extracellular vesicles (EVs) [16]. Besides, astrocytes are involved in maintaining ion and pH homeostasis in the central nervous system (CNS), providing glucose supply and antioxidant defense, and promoting the synthesis and clearance of neurotransmitters [13]. Thus, astrocytes have emerged as potential therapeutic targets for stroke [17].

### 1.3 Introduction to EVs and their role as potential therapeutic agents

EVs are nano-sized vesicles that carry various cargos: lipids, proteins, nucleic acids, and other substances [18, 19]. EVs with membrane lipid bilayers can also participate in tissue restoration and cross the BBB [20, 21]. Research has demonstrated that EVs contribute to cell information exchange, such as the interaction between neurons and glial cells [22]. EVs are implicated in the neurogenesis and angiogenesis of brain repair after stroke [23] and the treatment of CNS diseases [24]. However, the role and mechanism of EVs in ischemic stroke are still poorly understood and need to be further studied. Due to their small structural size, EVs are promising candidates for the targeted delivery of various materials to specific cells and have the ability to modify many ligands and receptors to interact with target molecules, as well as exhibiting good biocompatibility and functional homing ability [25]. Many researchers are now developing bioengineered EVs as a therapeutic approach for stroke. Incubation of EVs loaded with catalase or quercetin can provide neuroprotective effects in vitro and in vivo [26, 27]. Furthermore, treatment with curcumin-loaded EVs more effectively inhibited inflammatory response and apoptosis in the lesion area after cerebral ischemia in mice [28].

At the same time, bioengineered EVs do have many limitations, such as short circulation half-life, low payload, low targeting concentration, and rapid clearance from the lesion area [29]. Therefore, overcoming these shortcomings and further studies are warranted.

## 2. Ischemic stroke and the role of astrocytes

### 2.1 Pathophysiological processes involved in ischemic stroke

Ischemic stroke is caused by the occlusion of cerebral blood vessels, leading to hypoxia, glucose and lipid deficiency, which results in reduced ATP synthesis and energy deficiency, as well as impaired ion homeostasis and acid-base imbalance [30]. The pathophysiological changes caused by these dysfunctions include brain edema, inflammation, oxidative stress, neural cell death, and neurotransmitter disorders [31]. Recent advances in stroke research have highlighted key aspects of its pathogenesis, including cell excitotoxicity, inflammatory response, mitochondrial dysfunction, BBB damage, and cell death [31-33]. Given the complex pathophysiology of ischemic stroke, clearly understanding the accompanying injuries, and signaling mechanisms is the first key to developing therapies.

### 2.2 Importance of astrocytes in neuronal survival, neuroinflammation and blood-brain barrier integrity

As the abundant glial cells in the CNS, astrocytes provide nutrients to neurons, remove metabolites, maintain ion balance, and regulate synaptic activity [34]. When the brain undergoes ischemia, the loss of function of these astrocytes can seriously affect the survival of neurons. Astrocyte functions known to affect neuronal survival include glutamate uptake and release, the release of metabolic intermediates, free radical scavenging, and water transport [35]. For example, the re-release of lactate, alanine, citrate, and α-ketoglutarate from astrocytes can promote neuronal metabolism [35]. Astrocytes contain higher concentration of the antioxidant glutathione than neurons, which acts to resist reactive oxygen species from ischemic brain injury and to support neuronal survival [36]. Additionally, there is also evidence of ascorbate circulation in astrocytes and neurons, where dehydroascorbate easily passes the BBB and is converted to ascorbate, thus potentially mitigating ischemic brain damage [37]. Therefore, it is important to clarify astrocyte-neuron interactions during ischemia.

In addition, astrocytes can also protect the CNS from stress and pathological damage by transiently upregulating inflammatory processes. They enhance the inflammatory response by releasing anti-inflammatory cytokines transforming growth factor β (TGF-β) and chemokine CXCL12, thereby promoting the recruitment and proliferation of regulatory T cells (Tregs) at the site of injury [38]. However, excessive glial activation leads to more severe and chronic neuronal damage, ultimately propagating neuroinflammation and homeostasis imbalance [39].

Astrocytes have been identified as important mediators that modulate BBB formation and function. It has been demonstrated that astrocytes can increase the expression of tight junction related proteins, polarize the localization of transporters including P-glycoprotein (Pgp) and glucose transporter 1 (GLUT1), and activated specialized enzymes [40]. In addition, astrocytes also release chemical factors that transiently regulate endothelial permeability over a short period [41].

### 2.3 Astrocyte-mediated responses to ischemic stroke and their impact on post-stroke outcomes

During the early ischemic injury of the brain, astrocytes are activated, also known as reactive astrogliosis, which undergoes morphological changes of hyperplasia and hypertrophy, and increased expression of intermediate filament proteins including glial fibrillary acidic protein (GFAP), vimentin and nestin [17, 42]. Reactive astrocytes also produce and release free radicals (including NO, superoxide, and peroxynitrite), proinflammatory cytokines (IL-6, TNF-α, IL-1α and β, and interferon-γ) and chemokines (CCL2, CXCL1 and CXCL2), which can induce the death of neurons, as well as neurotoxicity mediators such as nitric oxide and increase the permeability of the BBB [43-46]. Several days after injury, astrocytes form glial scars around the ischemic infarction, creating physical and functional barriers. However, it has also been reported that reactive astrocytes can extensively express molecules that inhibit axonal regeneration, such as chondroitin sulfate proteoglycans (CSPGs), which leads to the failure of axon regeneration [47].

Astrocytes are also involved in the process of neuroprotection and repair. Astrocytic TGF-β is an important factor that serves to limit neuroinflammation [48]. As an anti-inflammatory and neuroprotective factor, TGF-β is upregulated at the chronic stage of stroke. Constructing an ischemic model using mice in which TGF-β signaling is specifically inhibited in astrocytes results in excessive neuroinflammation, larger infarct volume, and worse neurobehavioral outcomes [49]. Therefore, understanding and modulating astroglia-mediated responses to ischemic stroke is important for improving post-stroke outcomes. Finding therapeutic strategies that target astrocytes to diminish inflammation and promote neuroprotection and repair is essential. Such strategies can ultimately improve the prognosis of stroke patients.

## 3. EVS: Definition and characteristics

### 3.1 Overview of EVs and their biogenesis

EVs are double-membrane structure vesicles secreted by almost all cells and organisms [50]. In 1981, Trams found that the cultures from various normal and tumor cell lines shed vesicles with 5 '- nucleotidase activity. At the same time, they also found that microvesicles labeled with isotopes can be transferred to recipient cells. Therefore, they suggested that these vesicles with rich nutrients are involved in intercellular communication [51]. Pan and Johnstone first discovered vesicles in 1983 [52]. They reported that during the maturation of sheep reticulocytes, the release of transferrin receptors into extracellular space was associated with a small vesicle [19, 53, 54]. It is now well established that EVs have many roles in cell communication, which is facilitated by the transfer of cargo carried by EVs to the recipient cells [55].

### 3.2 Types of EVs, including exosomes, microvesicles and apoptotic bodies

According to the size and mode of formation, EVs are mainly classified into three types: exosomes (EXs) derived from endosomes with the size of 40-150nm, microvesicles (MVs) with sizes ranging from 100 to 1000nm that are shed by the plasma membrane, and apoptotic bodies with diameter ranging from 50 nm to 5000 nm and formed by the dissociation of the cell's plasma membrane from the cytoskeleton [56, 57]. EVs carry a variety of substances participating in intercellular communication, including lipids, proteins, nucleic acids and metabolites. The lipid composition of the EVs includes cholesterol (CHOL), sphingomyelin (SM), glycosphingolipids and phosphatidylserine (PS) [58]. It should be noted that EVs have significant effects on lipid metabolism, including lipid synthesis, transport and degradation [59]. The proteome of EVs includes membrane trafficking-related proteins, such as the tetraspanins (e.g., CD63, CD81, CD82, and CD9) [60] and integrins (e.g., ITGB1); cytosolic proteins, such as adapter (e.g., YWHAZ, YWHAE, SDCBP), tumor suppressor gene 101 (TSG101), and heat-shock proteins (e.g., HSP60, HSC70, HSP70, HSPA5, CCT2 and HSP90). Besides, extracellular proteins binding to EV membranes (e.g., A2M, ALB), Alix, LAMP-2, MHC class I and II proteins are found in EVs [61-63]. Thus, this set of proteins are often termed " Extracellular vesicular marker proteins." Nucleic acids carried by EVs include DNA, non-coding (nc) RNAs, and mRNA [64]. Lots of studies have demonstrated that EVs regulate the growth, differentiation and death of neighboring or distant cells by carrying microRNAs (miRNAs) [65]. In recent years, comprehensive databases like EVpedia and Vesiclepedia have been established to provide a comprehensive representation of EVs, documenting molecules (proteins, mRNAs, microRNAs, or lipids) observed within vesicles from different species, which suggests that EVs encompass an extremely broad and heterogeneous range of molecules [66, 67]. Based on these properties, EVs are being developed as therapeutic agents in a variety of disease models including ischemic stroke. In the following, the vesicles are referred to as EVs.

Isolation and identification of ADEVs from body fluids such as blood or cerebrospinal fluid (CSF) do face a set of challenges and bottlenecks. This is mainly due to the low concentration of ADEVs and the heterogeneity of EVs in the body fluids. Therefore, the isolation and identification of these ADEVs require highly sensitive techniques, as well as the identification of the morphology and markers of ADEVs. To address these challenges, strategies can be implemented to enhance the separation methods, such as ultracentrifugation and filter membrane separation, to improve the separation efficiency and purity of EVs. Improving detection sensitivity such as flow cytometry, electron microscopy, etc., to detect and identify EVs. In addition, amplification techniques such as PCR and next generation sequencing (NGS) can be combined to further increase detection sensitivity [68]. By simultaneously detecting multiple EVs surface or cell-specific markers, the origin of the EVs can be more accurately identified. For example, the presence of α-synuclein and clusterin carried by neuron-derived EVs in serum can serve as predictive and differentiating markers for Parkinson’s disease (PD), thereby aiding clinical judgment and targeted treatment [69, 70]. Common protein markers for ADEVs include the astrocyte-specific proteins excitatory amino acid transporter1 (EAAT1/GLAST) and glial fibrillary acidic protein (GFAP) [71]. Astrocytes have been shown to release EVs containing synapsin I, a glycoprotein capable of modulating neurite growth, when cultured with 75-80 mM potassium chloride (KCl) [72]. Additionally, astrocyte-derived EV content can be altered in response to pro-inflammatory cytokines. Chaudhuri and colleagues demonstrated that treatment with ATP, IL-1β, and TNF-α increased the expression of 7, 10, and 15 distinct miRNAs, respectively, in astrocyte-derived EVs relative to the untreatment control [73, 74]. At the same time, with the rapid development of advanced technologies, such as nanotechnology and single-cell analysis, isolation and identification efficiency of EVs were greatly improved.

### 3.3 Composition and cargo of ADEVs

Astrocytes secret matrix metalloproteinases (MMPs), including MMP-2 and MMP-9, some of which are released into extracellular space by vesicles to participate in the regulation of neuroinflammation [75]. MMPs are a family of zinc-dependent endopeptidases that are secreted or membrane-bound and regulate extracellular matrix, cytokines, chemokines, cell adhesion molecules, and plasma membrane receptors through proteolytic cleavage. It can be tightly regulated by specific endogenous tissue inhibitors of matrix metalloproteinases (TIMPs) to jointly regulate the cellular microenvironment under physiological and pathological conditions [76]. Research has indicated that amyloid peptide triggers the secretion of proapoptotic EVs associated with ceramide and prostate apoptosis response 4 (PAR-4) in astrocytes [77]. Mutant copper-zinc superoxide dismutase (SOD1) alterations in the protein secretion pathway of astrocytes. This results in the activation of unconventional secretory pathways, and ADEVs efficiently transfer SOD1 to spinal cord neurons and induce motor neuron death [78]. Proteomic analysis showed that the cargo carried by ADEVs also included neuroprotective proteins such as heat shock protein, alpha-synuclein, and lipoprotein receptor-related protein 1 (LRP1), as well as apolipoprotein E (APOE), which negatively regulates neuronal apoptosis, and peroxidase homolog, which supports neuronal oxidative stress management. There are proteins that promote neuronal excitability, such as potassium channel tetramerization domain containing 12 (KCTD12), kinesin family member 5B (KIF5B), glucose-6-phosphate dehydrogenase (G6PD) and spectrin-alpha non-erythrocytic1 (SPTAN1) [79]. Reports also underscore the roles of proteins found in ADEVs, such as valosin-containing protein and peptidyl-prolyl cis-trans isomerase A (Cyclophilin A) in amyotrophic lateral sclerosis (ALS) and frontotemporal lobar degeneration [80]. ADEVs also contain proteins associated with neuroprotection such as vascular endothelial growth factor (VEGF) and synapsin 1 [81]. Therefore, comprehensive analysis of the cargo carried by ADEVs could help us to understand the mechanism of ADEVs in neurological disorders and develop treatment for neurological diseases.

## 4. Cell interaction and communication through ADEVs in ischemic stroke

### 4.1 Interactions between ADEVs and neurons

#### 4.1.1 Delivery of neuroprotective factors and growth factors

The cargos and effects of ADEVs in stroke are listed in Table 1 and described below.

*FGF-2 and VEGF.* Studies have shown that ADEVs actively participate in cerebral angiogenesis, neurite regeneration, maintenance of neural homeostasis and neuroprotection after stroke [17, 82]. Proia et al. demonstrated that EVs secreted by astrocytes carry soluble factors such as basic fibroblast growth factor (bFGF or FGF-2) and VEGF. These factors can transfer information between cells by binding to their respective receptors [83]. A large number of studies have shown that FGF-2 actively participates in the proliferation, migration and differentiation of neural stem cells through FGFR signaling. FGF-2 also plays an important role in axonal and tissue regeneration. On the other hand, VEGF contributes to angiogenesis, neurogenesis, synapto-genesis, synaptic plasticity, and neuroprotection in the brain [84]. Therefore, for certain neurological diseases like stroke, exogenous administration of ADEVs containing FGF-2 and VEGF may hold therapeutic potential.

**Table 1:** The possible increased cargos by ADEVs after ischemic stroke.

Cargos	Main effects	References
**PrP**	Protects neuronal cells from oxidative stress, hypoxia, ischemia and hypoglycemia in the ischemic environment	Guitart et al. (2015)
**ApoD**	Mediate neuronal cell survival and functional integrity in response to oxidative stress	Pascua et al. (2018)
**FGF-2, VEGF**	FGF-2 is involved in the proliferation and migration of neural stem cells VEGF can promote cerebral angiogenesis, neurogenesis, synaptic plasticity, and neuroprotective activity	Proia et al. (2008)
**Synapsin I**	Protect neurons from ischemic damage and promote cell regeneration and survival under oxidative stress	Wang et al. (2011)
**Hsp70**	Change the microenvironment of astrocytes by regulating signal kinases under stress conditions such as hypoxia-ischemia, and have an impact on cell survival by transferring EVs to neighboring neurons	Taylor et al. (2007)
**NGB**	Have antioxidant, anti-apoptotic, and anti-inflammatory effects and may act as a neuroprotective agent against hypoxic/ischemic injury, β-amyloid, or H_2_O_2_ toxicity	Venturini et al. (2019)
**EAAT-1, EAAT-2**	Reduces extracellular glutamate concentrations and reduces excitotoxicity in the brain to maintain neural homeostasis	Gosselin et al. (2013)
**STI1**	High affinity binds to PrP^C^ in neurons and mediates calcium influx through alfa-7 nicotinic acetylcholine receptors, activation of PKA, ERK1/2 and PI3K, thereby regulating neuronal survival and differentiation and memory formation in vitro	Hajj et al. (2013)
**miR-125a-5p, miR-16-5p**	Downregulate neuronal dendritic growth and reduce cell survival	Chaudhuri et al. (2018)
**LCN2**	Induces neuronal loss and neurodegeneration	Liu et al. (2022)
**mitochondria**	Promote the recovery of damaged neurons after stroke	Hayakawa et al. (2016)

PrP, Prion protein; ApoD, Apolipoprotein D; WT, wild-type; ApoD-KO, Apolipoprotein D-Knock out; FGF-2, fibroblast growth factor; VEGF, vascular endothelial growth factor; HSPs, Heat shock proteins; NGB, neuroglobin; EAAT-1/2, excitatory amino-acid transporters-1/2; STI-1, stress-inducible protein 1; PrP^C^, cellular prion protein; LCN2, lipocalin-2.

*Heat shock proteins.* Heat shock proteins (HSPs) constitute a family of chaperone proteins, which can be upregulated in response to different cellular or environmental stress conditions. They can act as cell chaperones to promote proper protein folding and remove misfolded or aggregated proteins [85]. HSPs offer protective effects against various brain injuries, including stroke. It is known that Hsp70 is expressed at low levels in healthy brains. However, after cerebral ischemia, Hsp70 is mainly induced in neurons in the penumbra, and also expressed in microglia, astrocytes and endothelial cells near the infarct area [86]. Taylor and colleagues suggested that HSP70 could be carried by ADEVs and released to extracellular [87]. After cerebral ischemia, Hsp27 is expressed in astrocytes in the ischemic area [88]. Protein kinase D (PKD) phosphorylation induced by ischemia stimulates phosphorylation of Hsp27, then phosphorylated Hsp27 binds to ischemia-activated (phosphorylated) apoptosis signal-regulating kinase1 (ASK1) and prevents ASK1 signaling through MKK4/7, c-Jun NH (2)-terminal kinase (JNK) and c-Jun, thereby inhibiting apoptosis and reducing infarct volume of cerebral ischemia [86, 89]. Williams's team have reported that astrocytes exposed to amyloid-β (Aβ) release EVs carrying HspB1 (Hsp25/27), which could potentially exert a protective effect against neurodegeneration [90].

*Prion protein.* Prion protein (PrP) is a cell surface glycoprotein anchored by glycosylphosphatidylinositol and used as a sensor for oxidative stress. Current research has indicated that PrP possesses a protective effect under ischemic conditions. In the stroke mouse model, compared with the wild-type (WT) mice, the brain damage volume of PrP deficient (PrP KO) mice increased, and overexpression of PrP could worsen stroke outcomes [91]. In addition, Guitart et al. reported that EVs carrying PrP and other molecules (S3 and P0 proteins, apolipoprotein E, and laminin receptor dimer) released by astrocytes can improve neuronal survival under hypoxic and ischemic conditions [92].

*Apolipoprotein D.* Apolipoprotein D (ApoD), a neuroprotective protein, is upregulated in response to oxidative stress. It is transported by EVs derived from astrocytes and internalized by neurons. In vitro experiments proved that the knockout of ApoD increased astrocytes death when exposed to oxidative stress. However, the viability of astrocytes derived from ApoD knockout mice was enhanced when cultured in a medium containing ADEVs. These results indicate that ApoD-Containing EVs play a protective role by inhibiting the oxidative stress of glial cells, and mediating apolipoprotein-dependent neuroprotection [93].

*Neuroglobin.* In a separate study, Venturini et al. reported that ADEVs can transport neuroglobin (NGB) to neurons, which has antioxidant, anti-apoptotic and anti-inflammatory effects, and may be used as a neuroprotective agent against hypoxia/ischemia injury, amyloid protein or H_2_O_2_ toxicity. It was found that ADEVs released NGB selectively transported to neurons and participated in the beneficial role of astrocytes in ischemic injury [94].

#### 4.1.2 Modulation of neuronal survival, synaptic plasticity, and functional recovery

ADEVs have been attributed with pivotal roles in the central nervous system. Studies have shown that these EVs can have an impact on neuronal survival and axon remodeling by releasing vesicles containing biologically active molecules. Synaptic proteins promote neurite growth, neuronal survival, synaptic maturation, neurotransmitter release and transmission, and synaptic plasticity [84]. Wang et al. found that synapsin I, an oligomannose-carrying glycoprotein, was also detected in ADEVs when astrocytes were cultured in models of hypoxia/glucose deprivation or hydrogen peroxide-induced ischemia. Comparing the effect of EVs from wild-type or synapse protein deficient astrocytes confirmed that ADEVs protect neurons from ischemic injury, promote neuronal growth, and participate in regulating the interaction between glia and neurons via synapsin I [95].

Hajj et al. reported that stress-inducible protein 1 (STI1) is released from ADEVs and binds to cellular prion protein (PrP^C^) in neurons with high affinity. STI1, also known as heat shock protein-organizing protein (Hop), is demonstrated to enhance neuronal protein synthesis and promote neurite outgrowth, and it is thought to contribute to recovery after stroke [96]. This interaction results in calcium influx through alfa-7 nicotinic acetylcholine receptors, which activate PKA, ERK1/2 and PI3K, thereby regulating neuronal survival and differentiation and memory formation in vitro [97].

Gosselin and his colleagues found that ADEVs clear extracellular glutamate through membrane excitatory amino acid transporters (EAAT1 and EAAT-2), which play an important role in maintaining the stability of the CNS [98]. Excitatory amino acid transporter (EAAT) is a membrane transporter in neurons and astrocytes. It participates in glutamate reuptake, keeps extracellular glutamate at a low concentration, and reduces brain excitatory toxicity [99]. It is well known that in ischemic stroke, the increase of extracellular glutamate concentration or the imbalance of glutamate processing will lead to brain excitotoxicity and a large number of cell death [100]. This suggests that leveraging ADEVs' capability to clear glutamate could potentially serve as a therapeutic strategy to address this imbalance.

ADEVs could play a therapeutic role in ischemic stroke by carrying mitochondria. Studies have reported that, ADEVs contain mitochondria that transfer to damaged cortical neurons after ischemic stroke and would promote the recovery of neuronal dendrites and ATP levels [101]. Hayakawa et al. found that astrocytes could release mitochondria to damaged neurons through a calcium-dependent mechanism of CD38 and cyclic ADP ribose signaling after stroke, which could contribute to neurological protection and recovery after stroke [102]. A recent study found that stereotaxic injection of isolated healthy astrocyte mitochondria into the striatum of middle cerebral artery occlusion (MCAO) rats resulted in a significant reduction in cerebral infarct volume[103]. Mitochondria are essential organelles that regulate adenosine triphosphate (ATP) production, intracellular Ca^2+^ homeostasis, cell survival, and apoptosis [104]. Under stress conditions, they exert therapeutic effects in damaged cells by transferring EVs, gap junctions (GJs), and tunneling nanotubes (TNTs) [103]. Mitochondrial dysfunction is recognized as a hallmark of ischemic stroke and has been implicated in the pathology of ischemia and reperfusion [105]. Thus, isolation of ADEVs carrying mitochondria for transfer may be a novel strategy for the treatment of mitochondrial dysfunction after stroke.

#### 4.1.3 Modify the cargo to respond to changes in the inflammatory environment

Extracellular stimulation can alter the cargo composition and functions of ADEVs. Using microRNA expression analysis and quantitative proteomic analysis, Chaudhuri and colleagues performed the first comprehensive comparison of ADEVs in response to trophic (ATP), inflammatory (IL-1β and TNF-α), and anti-inflammatory (IL-10) stimulation. Compared with EVs released by IL-1β and TNF-α treated astrocytes (ADEV-IL-1β; ADEV-TNF-α), special neurite outgrowth-promoting proteins, such as Ribosomal Protein L10 (RPL10), neuropilin and tolloid like 1 (NETO1), were detected in EVs released by ATP-treated astrocytes (ADEV-ATP). In addition, IL-10 stimulates astrocyte shedding EVs (ADEV- IL-10) that also contain proteins with the same axonal guidance signal. Exposure of primary hippocampal neurons to ADEV-ATP or ADEV-IL-10 increased neurite length and complexity, and improved cell survival. On the other hand, ADEV-IL-1β and ADEV-TNF-α were more abundant and enriched with target proteins related to neurotrophic signaling than ADEV-ATP. The results further confirmed that miR-125a-5p and miR-16-5p enriched in ADEV-IL-1β and ADEV-TNF-α targeted neurotrophin receptor NTRK3 (TRKC) and Bcl2 to down-regulate neuronal dendritic outgrowth and cell survival. However, molecular interference of miR-125a-5p and miR-16-5p prevented this adverse effect. On the other hand, when primary hippocampal neurons are exposed to ADEVs following a trophic stimulus (ATP) or an anti-inflammatory stimulus (IL-10), this can increase the length and complexity of neurite, synaptic transmission and cell survival [73, 74]. Thus, when the microenvironment changes, astrocytes alter the miRNA and protein of EVs, and control the transcription and translation expression of the modified protein through miRNA, thereby targeting to modulate the synaptic stability and excitability of neurons.

After stroke, activated astrocytes not only provide neuroprotection but also adopt toxic functions, such as the release of inflammatory cytokines including interleukins (IL-6, IL-10, and IL-1β), IFN-γ, and TGF-β [13]. It has also been reported that reactive astrocytes secrete the lipocalin-2 (LCN2) glycoprotein to regulate cellular processes such as inflammation and injury in neurons after stroke [106]. In stroke patients and animal models, LCN2 is significantly elevated, proven to be related to neuroinflammation and cell death, and used as a biomarker for brain injury [107]. Liu et al. demonstrated for the first time that overexpression of Na^+^/H^+^ exchanger 1 (NHE1) in reactive astrocytes triggers NADPH oxidase (NOX) - nuclear factor κB (NF-κB) signaling and ROS production to induce upregulation of LCN2 expression. The release of ADEVs carrying LCN2 increases neuronal cell death and neurodegeneration. Blocking NHE1 activity in astrocytes was beneficial to reduce the secretion of LCN2-carrying ADEVs [108]. Thus, astrocytic NHE1 protein is a therapeutic target that could help reduce LCN2-mediated neurotoxicity after ischemic stroke.

### 4.2 Interactions between ADEVs and microglia

#### 4.2.1 Modulation of microglial activation, polarization, and phagocytic activity

During cerebral ischemia, microglia can be activated into two phenotypes: M1 microglia, which secrete proinflammatory cytokines (e.g., TNFα, IL-23, IL-1β, IL-12), exacerbating neuronal damage; M2 microglia, which exhibit anti-inflammatory responses and promote neuronal repair [109]. Astrocytes are the source of many chemokines and are involved in the M1 to M2 phenotype switching of microglia [110]. In addition, it has been reported that astrocytes can participate in regulating the activation, polarization, and phagocytic activity of microglia by secreting EVs. Astrocytes exposed to morphine released a large number of miR-138-carrying EVs, which were taken up by microglia and resulted in the binding of miR-138 motifs to endosomal toll-like receptor (TLR) 7, leading to the activation of microglia [111]. After spinal cord injury (SCI) activated astrocytes release C-C Motif Chemokine Ligand 2 (CCL2), which binds to C-C motif receptor 2 (CCR2) to induce microglia activation and neuronal apoptosis [112]. Hu et al. also found that ADEVs could be taken up by microglia under the stimulation of morphine based on in vivo and in vitro experiments, followed by upregulation of long intergenic noncoding RNA (lincRNA)-Cox2 expression, which eventually led to impaired phagocytosis of microglia. However, intranasal delivery of EVs containing RNA restored the phagocytic activity of microglia in morphine treated mice [113]. But at present, no specific studies have reported the communication and signaling between ADEVs and microglia after ischemic stroke.

#### 4.2.2 Regulation of neuroinflammatory responses and resolution of inflammation

Crosstalk between astrocytes and microglia plays an important role in neuroinflammation, but the mechanism mediates such interaction remains poorly understood. IL-10 derived from astrocytes alters the phenotype of microglia as well as lymphocyte recruitment to improve neural survival [114]. Han et al. found that dual immunoglobulin domain-containing cell adhesion molecules (DICAM) carried by ADEVs was involved in the inhibition of microglial activation and subsequent attenuation of neuroinflammation [115]. Some studies have found that miR-873a-5p is the most expressed miRNA in ADEVs in human traumatic brain tissue. In addition, ADEVs significantly inhibited LPS-induced M1 phenotype transformation of microglia and microglia-mediated neuroinflammation by reducing the phosphorylation of ERK and NF-κB p65 via miR-873a-5p, led to attenuation of neurological deficits after traumatic brain injury (TBI) [116]. In ischemic stroke, it has been reported that miR-223-3p loaded in mesenchymal stem cell-derived extracellular vesicles (MSC-EVs) can attenuate ischemic stroke-induced injury through inhibiting microglial M1 polarization mediated pro-inflammatory response [117]. MiRNA-126 carried by EVs secreted by adipose derived stem cells (ADSCs) inhibited the activation of microglia in the brain of stroke mice, reduced inflammatory response and increased neurogenesis and angiogenesis [118]. These studies suggest that ADEVS-derived miRNA could modulate the phenotype and function of microglia. However, more research is needed to explore the communication between ADEVs and microglia in ischemic stroke.

### 4.3 Interactions between ADEVs and endothelial cells

#### 4.3.1 Promotion of angiogenesis and vascular repair

As one of the important components of BBB, astrocytes can interact with endothelial cells to regulate the morphology of endothelial cells and angiogenesis [119]. Both astrocytic gene expression data analysis and in vivo two-photon imaging confirmed that astrocytes promote vascular repair and remodeling after ischemic stroke in mice [120]. Astrocytes can secrete VEGF to promote vascular growth and remodeling during embryogenesis. Although VEGF is a pro-angiogenic factor during development, in adulthood, VEGF reduces BBB integrity under inflammatory conditions [121]. It has long been confirmed that astrocytes can transport VEGF by shedding EVs [83]. Additionally, astrocytes secrete Sonic Hedgehog (SHh), which is involved in neuronal guidance and angiogenesis [122]. In addition, cerebral angiogenesis-related molecules such as miR-210 [123], miR-150 [124], IL-17A [125] and Dipeptidyl peptidase IV (DPPIV) [126] were confirmed to be highly expressed in astrocytes. However, whether ADEVs can also carry these cargoes and participate in vascular remodeling after cerebral ischemia is still unclear.

#### 4.3.2 Regulation of BBB integrity and permeability

Astrocytes constitute a significant component of the BBB and communicate with endothelial cells by secreting growth factors, morphogens, and EVs. These secreted factors control the integrity of the BBB by regulating tight junction proteins. Communication between astrocytes and endothelial cells is extremely important in neurological diseases that involve BBB damage [41]. Astrocytes play an indispensable role in the formation and maintenance of BBB, and dysfunction of astrocyte in the early stage of stroke may be a key factor in BBB injury [127]. In a chimeric BBB model consisting of human brain endothelial cells (hCMEC/D3) and wild-type astrocytes, treatment with wild-type ADEVs was found to increase trans-endothelial electrical resistance and upregulate tight junction proteins in hCMEC/D3, thereby supporting BBB integrity [128]. In addition, astrocytes also release angiotensin converting enzyme-1 (ACE-1), an enzyme that converts angiotensin I (Ang I) into angiotensin II (Ang II), which acts on type 1 angiotensin II receptor (AT1) expressed by endothelial cells. In the CNS, AT1 activation restricts BBB permeability and stabilizes connexin function by mobilizing into lipid rafts [129]. EVs play an important role in regulating BBB integrity and permeability after cerebral ischemia. MSC-EVs target TLR4 and deliver miR-125b-5p to inhibit astrocyte activation and inflammation, thereby attenuating the disruption of BBB integrity and alleviating hemorrhage in a t-PA induced ischemic stroke mice model [130]. Neural Progenitor Cell-Derived EVs enhance poststroke BBB integrity via regulating ATP-Binding Cassette Transporter B1 (ABCB1) and matrix metalloproteinase 9 (MMP-9), attenuating inflammatory cell recruitment by inhibition of the NF-κB pathway in stroke mice [131]. However, studies focusing on the mechanism of ADEVs' impact on BBB after stroke are still limited, necessitating further investigation.

### 4.4 Interactions between ADEVs and oligodendrocyte precursor cells

The excessive release of glutamate, oxidative stress and inflammation after ischemic brain injury can lead to demyelination. Demyelination is the main pathological manifestation of white matter injury (WMI), characterized by oligodendrocyte death and myelin loss, resulting in memory loss and cognitive impairment after ischemic stroke [132]. As a pool of migrating and proliferating adult progenitor cells, oligodendrocyte precursor cells (OPCs) distributed throughout the CNS and can differentiate and mature into oligodendrocytes to generate the myelin sheath that envelops axons [133]. Although OPCs located in the subventricular zone (SVZ) and corpus callosum (CC) can migrate to the surrounding area of ischemia, they are highly vulnerable in the hypoxic-ischemic environment, resulting in massive apoptosis and failure to successfully mature and differentiate into oligodendrocytes [134]. Therefore, the proliferation and successful differentiation of OPCs into mature oligodendrocytes stand as critical factors in the recovery from white matter damage and demyelination following stroke. Studies have pointed out that astrocytes participate in the maturation and differentiation of OPCs and play various roles in demyelination and remyelination. Xu et al. established a simulated ischemia model in vitro, which proved that ADEVs under mild hypoxia promoted the differentiation and migration of oligodendrocyte precursor cells, while ADEVs under severe hypoxia inhibited the proliferation of OPCs. This suggests that ADEVs may be a potential therapeutic target for the regeneration and repair of myelin sheath [135]. Similarly, further investigations are warranted to elucidate the interplay between ADEVs and oligodendrocytes, as well as the signaling pathways through which ADEVs contribute to white matter injury and demyelination after stroke.

### 4.5 Interactions between ADEVs and peripheral immune cells

ADEVs not only regulate neighboring cells within the CNS, but also facilitate interaction between the CNS and peripheral organs. Dickens et al. found that ADEVs rapidly enter the peripheral circulation and promote peripheral leukocyte migration by regulating acute cytokine response (ACR) in a mouse model of focal brain injury induced by intracerebral injection of interleukin-1β (IL-1β). Through bioinformatics analysis of the cargo carried by EVs and in vivo experiments in mice, they determined that ADEVs responded to IL-1β by inhibiting peroxisome proliferator-activated receptor α (PPARα), leading to increased NF-κB activity, which triggered the production of cytokines in the liver [136]. Discovering and expanding the mechanisms by which ADEVs regulate the communication between the brain and the peripheral immune system will lead to a major breakthrough in the treatment of ischemic stroke.

EVs derived from unstimulated/resting astrocytes, as well as those triggered by IL-10, exhibited a substantial reduction in IFN-γ and IL-17A levels. However, the level of IL-17A was increased in ADEVs stimulated with IL-10 and TGF-β [137]. Furthermore, ADEVs were found to exert regulatory effects on the release of CCL2 from T cell cultures. Studies have shown that CCL2 released by glial cells promotes the migration of monocytes and T cell nuclear dendritic cells to the CNS [138]. Moreover, exposure of astrocytes to proinflammatory cytokines (such as TNFα, IL-1a, C1q) has been linked to an escalated release of EVs. This, in turn, amplifies CCL2 production from infiltrating T cells, thereby promoting monocyte recruitment and increasing T cell recruitment to exacerbate the immune response [137]. However, the mechanism of immune cell infiltration mediated by ADEVs in ischemic stroke remains unclear. It is of great significance to study the role of ADEV in mediating the interaction between peripheral immunity and the central nervous system.

## 5. Therapeutic mechanisms of ADEVs in ischemic stroke

### 5.1 Neuroprotective effects of ADEVs in ischemic stroke

#### 5.1.1 Delivery of neuroprotective factors

Neuroprotective factors can protect neurons from injury and promote neural repair. Blocking transient receptor potential vanilloid 2 (TRPV2) in astrocytes has been reported to increase astrocyte proliferation, nerve growth factor (NGF) mRNA synthesis, and NGF secretion, which have neuroprotective effects in the early stages of stroke [139]. Zamanian et al. systematically analyzed the transcriptional databases of reactive astrocytes after LPS and MCAO injury models [140]. The reactive astrocyte phenotype was found to be strongly dependent on the injury type, and the astrocyte response to ischemia upregulated many neurotrophic genes, including the neurotrophic cytokines, leukemia inhibitory factor (LIF) and cardiotrophin-like cytokine factor 1 (CLCF1), brain-derived neurotrophic factor (BDNF), growth differentiation factor 15 (GDF15), etc., promoting neuronal survival, repair, and recovery. Mesencephalic astrocyte-derived neurotrophic factor (MANF) protects against endoplasmic reticulum stress-induced injury [141]. Besides, reactive astrocytes can secrete a variety of factors, including gliotransmitters (glutamate, ATP, and D-serine), glial cell line-derived neurotrophic factor (GDNF), that orchestrate effective communication within the microenvironment of neurons [142]. ADEVs play a pivotal role in facilitating communication between astrocytes and neighboring cells. However, direct evidence elucidating whether ADEVs can transport these factors to contribute to transmission following stroke is currently lacking.

#### 5.1.2 Modulation of oxidative stress and apoptosis

ADEVs play an important role in reducing inflammation and oxidative stress during cerebral hypoxic-ischemic injury. Astrocytes actively regulate immune responses [143]. In addition to participating in proinflammatory responses, they also contribute to maintaining the BBB integrity and reducing neuroinflammation by releasing EVs [144]. Delivery of miRNAs through EVs has emerged as a novel cellular communication mechanism, augmenting communication complexity between cells [145]. MiRNAs function as post-transcriptional regulators of gene expression, and are pivotal in brain development and various neurological diseases [146]. For example, miR-7a-2-3p can inhibit neuronal apoptosis after oxygen-glucose deprivation (OGD) [147]. Ischemic stroke is characterized by multi-stage and closely interconnected series of neuropathological reactions, including intense neuroinflammation, oxidative stress, BBB rupture and neuronal damage [148, 149]. Nonetheless, the exact mechanisms and pathways driving these processes remain elusive, underscoring the potential to elucidate neuronal protection mechanisms and further explore effective intervention strategies.

**Figure 1.**
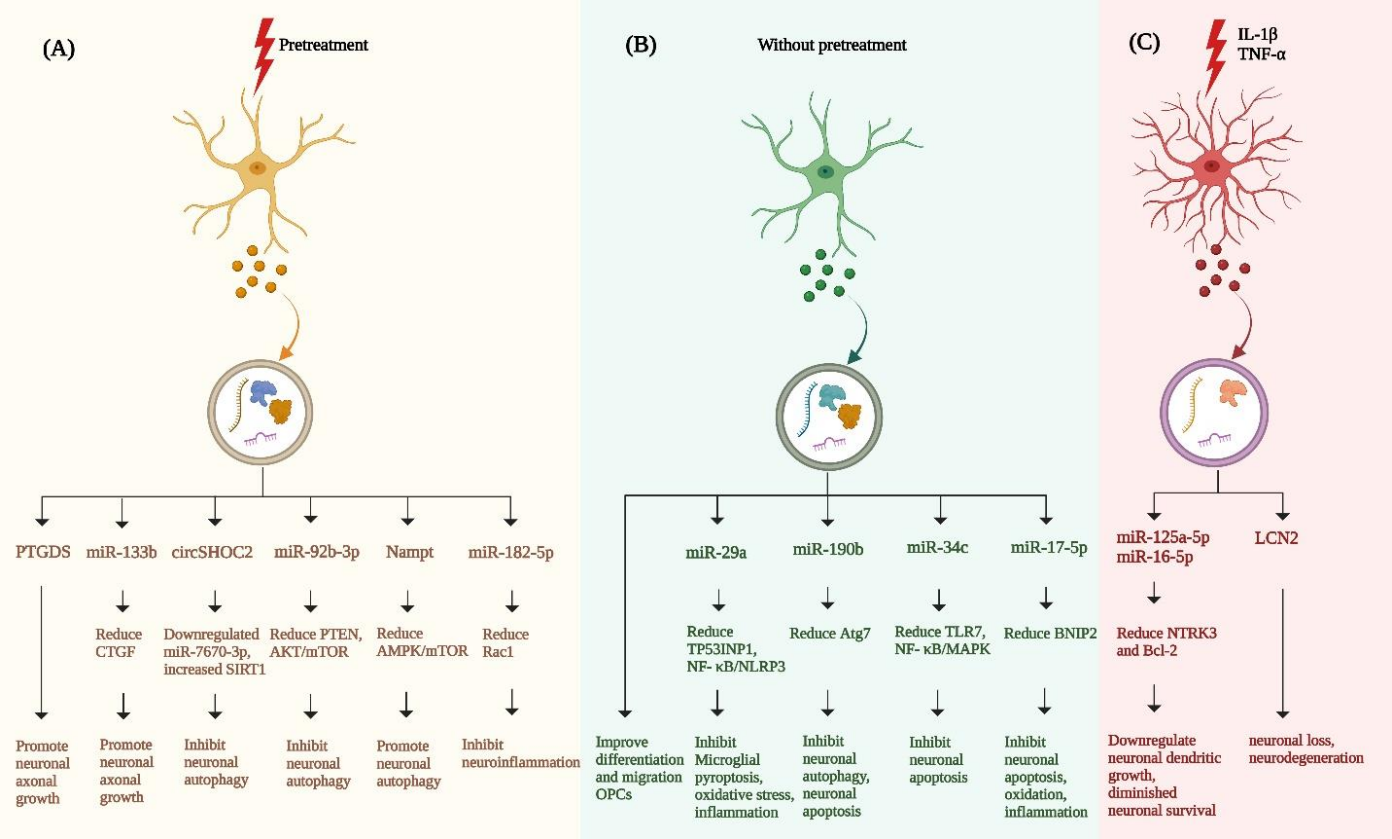
**Overview of the therapeutic mechanism and signaling pathways of astrocyte-derived EVs on ischemic stroke**. (**A**) After pretreatment (ischemic preconditioning or Sema3A-inhibitor treatment), (B) without pretreatment, (C) in the inflammatory factor (IL-1β, TNF- α) or the astrocyte under the environmental stimulation after stroke release EVs, which carry a large number of different molecules such as nucleic acids and proteins. EVs will transfer to damaged neurons, N9 microglia and OPCs, which regulate autophagy, apoptosis, axonal regeneration, oxidative stress and inflammation of neurons through various signal pathways, and reduce oxidative stress, scorch and inflammation of N9 microglia, and promote OPCs differentiation and migration. Therefore, ADEVs may be one of the important directions of drug development for ischemic stroke in the future.

MiR-34c is an important tumor suppressor and plays a key role in tumorigenesis, invasion and migration [150]. It was observed that miR-34c was significantly downregulated in the plasma of ischemic stroke patients [151]. Through both in vivo and in vitro models of cerebral ischemia-reperfusion (I/R) injury, Wu et al. confirmed that ADEVs treatment reduced neurological deficits, infarct volume, brain edema, inflammation, and neuronal damage in MCAO rats. In addition, miR-34c transported by ADEVs was found to target Toll-like receptor (TLR7) and down-regulate the NF-κB/MAPK axis, thereby reducing I/R-induced neuronal injury and expression of pro-inflammatory factors, promoting cell proliferation and inhibiting cell apoptosis after OGD/reperfusion (OGD/R)R treatment (Fig. 1) [152]. This study provides novel insights into potential therapies for cerebral I/R injury. Through in vivo and in vitro experiments, Du's team verified that miR-17-5p enriched ADEVs negatively regulated the expression of BNIP-2 in neurons under ischemic conditions, thus improving neurobehavior recovery and reducing infarct volume, neuronal apoptosis, oxidation stress (reduction of SOD, GSH-Px, CAT and induction of MDA) and inflammation (Fig. 1) [153]. The activation of miR-17-5p attenuated neuronal apoptosis and improved neurobehavior recovery following cerebral hypoxia and ischemia in rat pups [154]. As a homologous domain protein in the Bcl-2 family, BNIP-2 plays an important role in mitochondrial-mediated apoptosis [155]. BNIP-2 has been proven to be a proapoptotic factor in estrogen neuroprotection [156]. However, further research is required to fully comprehend the extent of miR-17-5p enrichment in ADEVs. These discoveries contribute new evidence for the utilization of extracellular vesicle-carried miRNAs in the realm of neuroprotection.

### 5.2 Modulation of neuroinflammation by ADEVs

#### 5.2.1 Regulation of inflammatory cytokines and chemokines

Communication between astrocytes and neurons plays a pivotal physiological role in the CNS. Astrocytes exert diverse effects on neurons encompassing structural, metabolic, and functional aspects, with outcomes ranging from neurotoxicity to neuroprotection. As mentioned above, astrocytes can exert neuroprotective effects by expressing neuroprotective factors such as NGF. In addition, their neurotoxic effects are mediated by the aging-related secretory phenotype (SASP), involving the release of proinflammatory cytokines such as IL-6 [157]. Reactive astrocytes also produce and release inflammatory cytokines (e.g., interleukins, TNF-α, TGF-β), chemokines (e.g., CXCL12), which induce neuronal death and neurotoxicity and increase the permeability of the BBB [142]. However, it has been reported that hypoxia and berberine pretreatment of ADEVs can reduce neuronal damage by inhibiting neuroinflammation.

The neuroprotective effects of astrocytes and EVs mediated by ischemic preconditioning (IPC) have been widely observed. Under mild ischemic preconditioning, astrocytes release neurotransmitters such as ATP [158] and glutamate [159], which function as sensors for ischemic tolerance. These neurotransmitters provide neuroprotection when confronted with subsequent episodes of severe ischemia [160]. Besides, studies showed that P2X7Rs was upregulated in astrocytes after ischemic preconditioning, and P2X7Rs can increase the target molecules of hypoxia-inducible factor-1α (HIF-1α) and induce the expression of neuroprotective molecules such as erythropoietin, thus inducing ischemic tolerance [161]. Furthermore, Li et al. demonstrated for the first time that remote IPC protects against brain ischemia by inducing the secretion of plasma EVs containing HIF-1α to enhance brain hypoxia tolerance [162]. Subsequent studies have also found cerebral-IPC significantly increased the release of plasma EVs containing miR-451a to alleviate ischemia-reperfusion (I/R) injury [163]. IPC with transient and sublethal ischemic attacks can increase the brain's resistance to subsequent severe ischemic injury [164], the protective mechanism may be related to the involvement of ADEVs.

Ischemic-preconditioned ADEVs (IP-ADEVs) have demonstrated enhanced neuroprotective capabilities compared to those from untreated astrocytes. Xu et al. found that EVs in the culture medium of astrocytes pretreated with sublethal OGD could be taken up by neurons and ameliorate OGD-induced neuronal damage. Besides, they found that miR-92b-3p levels were increased in IP-ADEVs, which exhibited a protective effect on OGD-treated neurons [65]. Therefore, they propose that IPC-mediated neuroprotection may be related to the transfer of IP-ADEVs-derived miR-92b-3p to neurons. To investigate whether miR-92b-3p also has neuroprotective effects in animals, Xu and colleagues successfully constructed a cerebral ischemic preconditioning model through MCAO. The results showed that IPC could alleviate the subsequent neurological damage caused by prolonged ischemia and stimulate the expression of miR-92b-3p in brain tissue, which reduced the expression of PTEN in neurons and subsequently activated Akt/mTOR signaling pathway [165]. This also suggests that IP-ADEVs can alleviate cerebral ischemia injury, and its mechanism may be related to the regulation of PTEN/Akt/mTOR signaling pathway by ADEVs-delivered miR-92b-3p (Fig. 1). Furthermore, Chen et al. found that IP-ADEVs had neuroprotective effects in vivo and in vitro. Compared with the PBS-treated ADEVs group, the IP-ADEVs treatment group significantly reduced OGD-induced neuronal cell apoptosis, decreased the levels of apoptosis-related proteins caspase-3 and Bax, and markedly promoted the expression of Bcl-2 [166]. In addition, the levels of tumor necrosis factor (TNF) -α, interleukin-6 (IL) -6, and IL-1β in neurons were reduced after treatment with IP-ADEVs compared with the PBS group, as measured by enzyme-linked immunosorbent assay (ELISA). The same conclusion was also reached by in vitro experiments. IP-ADEVs treatment greatly reduced infarct volume and neurobehavioral deficits. Furthermore, they demonstrated that circSHOC2 in IP-ADEVs suppressed neuronal apoptosis and ameliorated neuronal damage by regulating autophagy and acting on the miR-7670-3p/SIRT1 axis, which might provide strategies for the future treatment of ischemic stroke (Fig. 1).

Berberine is an alkaloid extracted from Coptis chinesis or other medicinal herbs, which has an antibacterial effect [167]. In recent years, berberine has been extensively studied and found to have neuroprotective effects in ischemic stroke [168], Alzheimer's disease (AD) [169] and other brain diseases. Ding et al. explored the effect and regulatory mechanism of berberine preconditioned ADEVs (BBR-ADEVs) and EVs derived from OGD/R pretreated astrocytes (OGD/R -ADEVs) on ischemic stroke. By establishing in vivo and in vitro ischemic models, BBR-ADEVs and OGD/R-ADEVs were found to improve OGD/R-induced neuronal apoptosis. It reduced the expression of IL-1β, IL-6 and TNF-α, reduced neuronal damage in vitro and in vivo, and inhibited neuroinflammation. However, BBR-ADEVs exhibited superior efficacy. High-throughput sequencing results showed that miR-182-5p was highly expressed in BBR-ADEVs, and the EVs transferred to the injured neurons and downregulated the expression of Rac1, thereby inhibiting neuroinflammation and attenuating neurological damage after ischemia [170].

The above research proves that compared with untreated ADEVs, IP-ADEVs exhibit enhanced capacity to mitigate neuronal damage resulting from ischemia. Moreover, many studies have found that even without pretreatment, ADEVs may improve neurobehavioral recovery after stroke [171], including reducing apoptosis and proinflammatory factors after stroke through modulating autophagy, or promoting axonal regeneration and functional repair after stroke (Table 2).

**Table 2:** Summary of the mechanism and signaling pathways of ADEVs in the treatment of ischemic stroke.

Species	Model	Interacting cells	Source of AS	Cell status	Cargos	Effect	Routes, dose, and timing of administration	Signaling pathways	Ref..
**SD rats**	MCAO, OGD	Neurons	cerebral cortices	Sema3A-I	PTGDS	Promote axonal growth	-	Sema3A/Rnd1/R-Ras/Akt/GSK-3β	Hira et al. (2018)
**mice**	MCAO	Neurons	-	reactive	miR-133b	Promote axonal growth	-	miR-133b/pikfyve	Liu et al. (2019)
**SD rats**	MCAO, OGD	Neurons	cerebral cortices	IP	miR-92b-3p	Inhibit autophagy	-	miR-92b-3p/PTEN/Akt/mTOR	Xu et al. (2017,2019)
**C57BL/6 mice**	MCAO, OGD	HT-22 cell	cerebral cortices	Normoxic	-	Inhobit autophagy	IV injection of 40μg/mL of EVs 1h after ischemia	-	Pei et al. (2019)
**C57BL/6 mice**	OGD	HT-22 cell	cerebral cortices	Normoxic	miR-190b	Inhibit autophagy	-	*miR-190b/Atg7*	Pei et al. (2020)
**C57BL/6 mice**	MCAO, OGD	Neurons	cerebral cortices	IP	circSHOC2	Inhibit autophagy	IV injection of three times a day for a total of 100 mg of EV per day after ischemia	circSHOC2/miR-7670-3p/SIRT1	Chen et al. (2020)
**C57BL/6 mice**	MCAO/R, OGD/R	Neurons	cerebral cortices	OGD/R	Nampt	Promote autophagy	IV injection of 20 μg/mL of EVs post MCAO/R	Nampt/AMPK/mTOR	Deng et al. (2022)
**Wistar rats**	cerebral I/R, OGD/R	N2a cell	purchase	Normoxic	miR-34c	Inhibit neuronal apoptosis	IV injection of 20 μg/mL of EVs after ischemia	miR-34c/TLR7/NF-κB/MAPK	Wu et al. (2020)
**rats**	MCAO, OGD	N9 microglia	cerebral cortex	Normoxic	miR-29a	Inhibit oxidative stress	IV injection of 50 μg/mL of EVs 24 h before MCAO	miR-29a/TP53INP1/NF-κB/NLRP3	Liu et al. (2021)
**SD rats**	HIBD, OGD	H19-7 cell	cerebral cortex	Normoxic	miR-17-5p	Inhibit oxidative stress	IP injection of 2-3 μg EVs 24 h before HIBD	miR-17-5p/BNIP-2	Li et al. (2021)
**SD rats**	OGD	OPCs	cerebral tissue	Normoxic, mild hypoxia, severe hypoxia	-	Promote diferentiation and migration of oligodendrocyte precursor cells	-	-	Xu et al. (2021)

#### 5.2.2 Modulation of microglial activation and polarization

Activated astrocytes can regulate microglial polarization by releasing miRNA transported by EVs in vitro and in vivo. Microglia are the most common and innate immune cells in the central nervous system. In ischemic stroke, microglia are activated and polarized into proinflammatory and anti-inflammatory phenotypes, initiating inflammatory and immune responses [172]. Studies have shown that miR-138 levels are upregulated in ADEVs stimulated by morphine, and this miRNA can be internalized by microglia, leading to microglial activation [111]. Recent evidence has shown that ADEVs enriched with miR-873a-5p significantly facilitate the transition of microglia towards an anti-inflammatory phenotype after traumatic brain injury by decreasing the phosphorylation of ERK and NF-κB p65 and attenuate microglia-mediated neuroinflammation and brain injury [116]. By establishing an OGD model and a BIR rat model, Li et al. found that the miR-29a carried by ADEVs downregulated tumor protein 53-induced nuclear protein 1 (TP53INP1) and NF- κB/NLRP3 pathway, reduced OGD-induced pyroptosis, oxidative stress, and inflammation of N9 microglia. In vivo experimental results corroborated these findings. Through this specific molecular pathway, ADEVs exhibited therapeutic effects on BIR rats, evident in the inhibition of proinflammatory factors in brain tissue and the improvement of neural function [173]. However, our understanding of the communication between astrocytes and microglia via EVs following stroke remains relatively limited and warrants further investigation.

#### 5.2.3 Modulation of autophagy in neurons by ADEVs

ADEVs are involved in regulating autophagy-related signaling pathways in vitro and in vivo, thereby inhibiting neuronal apoptosis and the production of pro-inflammatory factors caused by ischemic stroke. Autophagy is a highly conserved catabolic pathway, through which long-lived or misfolded proteins and damaged organelles are degraded into metabolic components that can be recycled to maintain homeostasis and normal cellular functions [174]. Accumulating results showed that autophagy was involved in the pathophysiological changes of ischemic stroke, but the role of autophagy in cerebral ischemia is controversial. Some studies suggest that autophagy can be detrimental, while others propose its potential neuroprotective effects [175]. Whether autophagy is beneficial or harmful depends on the rate and duration of its induction. There is increasing evidence that autophagy is activated in various cell types of the brain during ischemic stroke, including neurons, glial cells and microvascular cells [176]. Autophagy related signaling pathway is a promising target for the treatment of ischemic stroke, but the exact role of autophagy in ischemic events and its potential value in drug therapy remains to be confirmed [176].

Several studies have suggested the detrimental role of autophagy in ischemic stroke. Pei et al. first showed that ADEVs could inhibit OGD-induced neuronal apoptosis in vitro. Besides, ADEVs were shown to protect neurons from OGD-induced damage by inhibiting autophagy. Subsequent animal experiments also confirmed that ADEVs can inhibit the autophagy of neurons, thereby enhancing neuronal resilience against ischemic stress [22]. However, the mechanism by which ADEVs regulate autophagy remains unclear. The team continued to explore the mechanism of ADEVs regulating neuronal autophagy. Further in vitro studies confirmed that miR-190b in ADEVs was higher than that in astrocytes. It was identified that miR-190b carried by ADEVs could target the 3′-UTR region of the downstream neurotropic gene Atg7 to inhibit OGD-induced neuronal autophagy and attenuate neuronal apoptosis [177]. The mechanism of this effect is at least partially related to the transfer of miR-190b to neurons, resulting in autophagy inhibition. This later mechanism has been briefly described previously. Chen et al. confirmed that the protective mechanism of IP-ADEVs in ischemic stroke was associated with transferring circSHOC2 to neurons, and circSHOC2 promoted the expression of downstream target gene SIRT1 by inhibiting autophagy and sponging miR-7670-3p to promote neuronal survival and improve cell damage [166]. Numerous studies have highlighted the significant role of exonic circular RNAs (circRNAs) in modulating autophagy [166, 178].

On the other hand, autophagy assumes a protective role in ischemic stroke. Yang et al. observed the neuroprotective effect of ADEVs in acute ischemic stroke (AIS) through in vitro and in vivo experiments. It was found that after OGD/R induction, ADEVs release nicotinamide phosphoribosyltransferase (Nampt, also known as pre-B cell colony enhancing factor or visfatin), which is transferred to neurons. Then, Nampt leads to an upregulation of p-AMPK protein levels followed by a decrease of p-mTOR levels in neurons. Inhibition of p-mTOR further enhanced autophagy and reduced cell death in neurons [179]. It's worth noting that the temporal dynamics of autophagy after ischemic stroke exhibit a peak around 24 hours post-stroke. Under physiological and mildly stimulating conditions like transient ischemia, a moderate elevation in autophagy levels constitutes a crucial defense mechanism that neurons deploy to counter stressors [180]. However, excessive activation of autophagy leads to non-programmed cell death. Therefore, the balanced regulation of autophagy after stroke is very important.

### 5.3 Promotion of angiogenesis and neurovascular remodeling by ADEVs

#### 5.3.1 Delivery of pro-angiogenic factors

It has been previously reported that ADEVs can release VEGF and FGF-2 factors, which exert pro-angiogenic, neurogenic and synaptogenic effects. However, in vitro BBB models have shown that ischemic neurons induce astrocyte-derived VEGF production, leading to endothelial barrier damage accompanied with loss of tight junction proteins OCLN and CLN-5 [181]. Therefore, it is crucial to understand and modulate the dual role of VEGF conveyed by ADEVs in ischemic stroke [182]. In addition, it has been reported that astrocytes also express various factors including platelet derived growth factor (PDGF), TGF-β, matrix metalloproteinases (MMPs), etc, which stimulate the proliferation and migration of vascular smooth muscle cells and participate in vascular reconstruction and repair, [183, 184]. Studying whether ADEVs carry these cargoes and the underlying mechanisms could offer valuable insights into enhancing vascular repair following stroke.

#### 5.3.2 Induction of endothelial cell proliferation and migration

ADSC-EVs contain angiogenic proteins, including interleukin-8 (IL-8), CCL2, TIMP-1, TIMP-2, and vascular endothelial growth factor-D (VEGF-D). ADSC-EVs can increase the proliferation, migration, total vessel length and junction density of endothelial cells in vitro [185]. Endothelial progenitor cells-derived EVs (EPC-EVs) accelerated endothelialization at the early stage after carotid artery endothelial injury in rats and enhanced endothelial cell proliferation and migration in vitro [186]. EVs from human umbilical cord mesenchymal stem cells (hucMSCs-EVs) promote the proliferation, migration and angiogenesis of endothelial cells in a dose-dependent manner, which is mediated by Wnt4-induced β-catenin activation in endothelial cells [187]. At present, there is a lack of reports exploring the impact of ADEVs on the proliferation and migration of endothelial cells after stroke, which is one of the directions that needs to be addressed.

### 5.4 Regulation of neuronal plasticity and functional recovery by ADEVs

#### 5.4.1 Promotion of neurogenesis and synaptogenesis

Increasing evidence suggests that EVs from stem cells enhance neurogenesis, angiogenesis, and axon outgrowth while inhibiting inflammatory response, thereby enhancing functional recovery after stroke. EVs from human iPSC-derived neural stem cells are enriched in miRNAs and proteins involved in neuroprotection, anti-apoptosis, anti-oxidation and anti-inflammation. Moreover, these EVs contain miRNAs and proteins that facilitate synaptogenesis, synaptic plasticity, and cognitive functionality [188]. Among the extensively studied EVs are those derived from MSCs. When injected intravenously after transient MCAO, MSC-EVs have shown the potential to improve neurological function through the transport of specific miRNAs and molecules, thereby increasing neurogenesis, angiogenesis, axon growth and synaptogenesis [189, 190]. MiR-133b in MSC-EVs can serve as a key player in promoting neurogenesis and axonal remodeling [191]. The accumulating evidence strongly highlights the effectiveness of EVs as powerful facilitators of intercellular communication. EVs play a central role in diverse neurodevelopmental processes, including neurogenesis, synaptogenesis, and gliogenesis. As such, it becomes imperative to engage in comprehensive investigations to unravel the specific contributions of astrocyte-derived EVs in both normal and pathological neural development.

#### 5.4.2 Enhancement of axonal sprouting and regeneration

ADEVs are involved in axon maintenance and neural network plasticity [192]. Stroke can trigger astrocyte proliferation, resulting in alterations in astrocyte molecular expression and morphology. In severe cases, astrocytes participate in the formation of glial scars [193]. Because the glial scar provides a physical barrier for axon growth and expresses various axon growth inhibitors, the glial scar is considered to be harmful to neural recovery after stroke [194]. But astrocytes also play beneficial roles after stroke, such as improving synaptic function [195]. EVs are also involved in mediating axon growth. It has been reported that EVs derived from ischemic cerebral endothelial cells (isCEC-EVs) promote axon outgrowth by altering the distribution of miRNAs and their target proteins in recipient neurons [196]. Stroke-induced angiogenesis and axonal remodeling are one of the key factors in the brain repair process after stroke [197]. However, the direct effect of ADEVs on axonal growth after ischemia and the underlying molecular mechanism is still unclear.

In the subacute phase, Hira et al. [192] found that inhibition of semaphorin 3A (Sema3A) in the peri-infarct area promoted axonal regeneration and inhibited astrocytes activation, thus promoting functional recovery after stroke. Semaphorins are a large class of signal molecules and axon-oriented molecules, which are composed of secretory or membrane-bound proteins. Sema3A can induce the collapse of growth cones after CNS injury and is an extracellular matrix molecule that inhibits axon growth [198, 199]. Moreover, in vitro results showed that Sema3A inhibitor (Sema3A-I) decreased Rho family GTPase 1 (Rnd1) and increased R-RAS in neurons under OGD treatment, thereby phosphorylating Akt and glycogen synthase kinase 3β (GSK-3β). Increased phosphorylation of GSK-3β in axons, in turn, caused phosphorylation of high-molecular-weight neurofilament -immunoreactive axons after OGD. Furthermore, Sema3A-I inhibited OGD-induced astrocyte activation in cultured astrocytes. Sema3A-I-treated ischemic ADEVs further promoted axon elongation and Prostaglandin D_2_ Synthase (L-PGDS, also known as PTGDS) expression. L-PGDS can form Prostaglandin D2 (PGD2), which is the most abundant prostaglandin produced in the mammalian brain and is involved in many CNS diseases, such as multiple sclerosis [200, 201]. Moreover, L-PGDS has a protective effect on experimental transient and permanent cerebral ischemic mice models [202]. These results suggest that not only the neuronal Rnd1/R-Ras/Akt/GSK-3β signaling pathway, but also ADEVs may be potential therapeutic targets to promote axon regeneration and stroke recovery (Fig. 1).

Xin et al. showed that multipotent MSCs mediate the transfer of EVs carrying miR-133b to astrocytes and neurons. ADEVs enriched in miR-133b were shown to decrease the expression of connective tissue growth factor (CTGF), reduce glial scar wall, and induce neurite growth, thereby promoting neural recovery after stroke [191, 203]. Several studies have found that miR-133b actively participates in the neural axon development pathway and down regulates the expression of CTGF [204]. Glial scarring, a result of extracellular matrix deposition and astrogliosis, constitutes a significant impediment to axonal regeneration [205]. At this time, reactive astrocytes exhibit substantial CTGF expression, which acts as a major inhibitor of axonal growth within the injured region of the mammalian central nervous system; its expression also increases in the brain post-stroke [206]. Another study showed that exposure to hypoxia and hypoglycemia was found to trigger the overexpression of miR-133b in MSCs-EVs, thus promoting the release of ADEVs. As a secondary messenger, ADEVs significantly increased the number of axon branches and the total axon length of primary cortical neurons. In addition, Furthermore, ADEVs demonstrated a superior capability to promote neurite growth and plasticity compared to MSC-EVs, which indicates that the stimulated secondary release of ADEVs is related to the beneficial effect of MSC-EVs after stroke [207, 208]. Liu et al. demonstrated that overexpression of miR-133b in reactive astrocytes in vivo would enhance the release of EVs, it also increased the amount of miR-133b carried by these EVs, thereby promoting neurite remodeling and functional recovery after stroke (Fig. 1) [209].

## 6. Delivery strategies for aDEVs

### 6.1 Routes of administration for ADEVs in ischemic stroke therapy

#### 6.1.1 Intravenous injection

The administration routes for EVs in stroke treatment can be divided into systemic and local administration. Systemic administration includes intravenous injection via the tail, femoral vein or internal jugular vein, intra-arterial injection via the common carotid artery, as well as intraperitoneal and intranasal administration [210]. Among these options, intravenous injection of EVs is a commonly employed treatment approach. Injection of M2 microglia-derived EVs into the tail vein of mice from day 1 to day 7 after MCAO revealed that they were enriched in miR-124, which reduced the expression of astrocyte proliferation genes signal transducer, activator of transcription 3, GFAP, and Notch 1, and promoted the transformation of astrocytes into neuronal progenitors [211]. In addition, the EVs also reduced glial scar formation and promoted recovery after stroke. Bone marrow mesenchymal stem cell-derived EVs (BM-MSC-EVs) have been shown to be effective for TBI repair by intravenous or in situ injection [212]. However, achieving sustained delivery or aggregation of BM-MSC-EVs at the lesion site is still an obstacle for the treatment of TBI. Intravenous injection of BM-MSC-EVs can reduce inflammation, apoptosis, oxidative stress, and provide neuroprotection in the doxorubicin (DOX)-induced rat chemical brain model by regulating Wnt/β-catenin and hedgehog signaling pathways [213]. Table 2 summarizes the delivery route of ADEVs in rodent models of ischemic stroke, and it can be seen that intravenous injection is the most commonly used method, followed by intraperitoneal injection.

#### 6.1.2 Intracerebral injection

Injecting neural stem cell-derived extracellular vesicles (NSC-EVs) into the lateral ventricle of rats within hours after MCAO reduced lesion volume, microglia proliferation and apoptosis, and increased neuronal survival as measured 7 days post-administration [214]. Intracerebral administered neuroblastoma-derived EVs can carry glycosphingolipids (GSLs) to clear Aβ, which could provide a novel therapeutic intervention for AD [215]. Intracerebral injection of BM-MSC-EVs into APPswe/PS1dE9 AD mice at 3 and 5 months of age reduced Aβ plaque load and dystrophic neurites number in the cortex and hippocampus, which plays a potential role in the early stages of AD [216]. Currently, there is limited research regarding intraventricular injection of ADEVs for stroke treatment. Given the diverse delivery methods and their potential impacts on treatment outcomes, further investigation and comparative studies are necessary to better understand the benefits and limitations associated with different administration routes for ADEVs in stroke therapy.

### 6.2 Challenges and considerations in ADEVs delivery to the ischemic brain

#### 6.2.1 Blood-brain barrier penetration

Despite significant progress in drug delivery, effectively treating CNS diseases remains a considerable challenge, largely due to the intricate barrier posed by the BBB. This barrier protects CNS from harmful substances and reduces excessive immune responses [217]. More recently, EVs have emerged as a highly promising vehicle for targeted drug delivery in the brain. Chen and colleagues found that EVs could cross brain microvascular endothelial cell (BMEC) monolayer under TNF-α-stimulated inflammatory conditions, but not under normal conditions. Moreover, they also found that most of the exosomes were internalized by BMECs through transcellular pathways including endocytosis, MVB formation and exocytosis, and rarely through the paracellular route [218]. Furthermore, it has been shown that EVs can be designed to carry small molecules and proteins to cross the BBB and exert therapeutic effects. Brain endothelial cell-derived EVs can deliver anti-cancer drugs such as paclitaxel and doxorubicin across the BBB and into the brain, significantly reducing tumor growth [219]. The catalase-loaded exosomes (exoCAT) successfully crossed the BBB, provided neuroprotection and attenuated PD progression [220]. EVs secreted by CNS cells and immune cells, including neurons, astrocytes and microglia, may bypass BBB to exert their effects [218]. Published data suggest that EVs can cross the BBB, but the mechanism of interaction between ADEVs and the BBB remains unelucidated and requires further investigation.

#### 6.2.2 Optimization of dosage and timing

As preclinical data suggest, the dosage and timing of EVs administration are important factors in optimizing EVs-based therapy [221]. Currently, MSC-EVs administered at a dosage of 100μg have shown to provide long-term brain protection, gray and white matter repair, and increased neurogenesis and angiogenesis, as well as immunomodulation in experimental animal models of stroke [222]. However, the potential impact of a higher EV dosage on recovery remains uncertain. By using an in vitro hypoxia and glucose deprivation (OGD) model and an in vivo model of subcortical stroke rats, dose-response studies of EVs from adipose tissue-derived MSCs identified a dosage of 50μg MSC-EVs as the minimum effective dose to enhance protection and brain functional recovery from subcortical ischemic stroke [223]. At present, there are no detailed clinical trials on the dose and timing of ADEVs delivery. Table 2 summarizes the delivery does, and timing of ADEVs in rodent models of ischemic stroke. Despite the potential advantages of using ADEVs as a stroke treatment, the ideal dose, delivery method, and timing warrant thorough exploration and standardization to ensure both safety and efficacy. Engaging in further research and conducting clinical trials will undoubtedly contribute to addressing these critical issues and propelling the development of this promising treatment avenue.

### 6.3 Novel approaches for targeted and efficient delivery of ADEVs

#### 6.3.1 Surface modification for targeted delivery

EVs membrane and cell membrane have similar structures and contents, which can be modified for targeted drug delivery. This approach enables enhanced drug accumulation at specific sites while mitigating potential side effects. To date, effective methods to improve the targeting ability of EVs include chemical modification and genetic engineering [224]. Binding various ligands such as peptides, antibodies, vitamins and carbohydrates to the surface of EVs can improve their targeting ability [225]. For example, Ye et al. developed EVs containing a multifunctional peptide as a targeting ligand modified with methotrexate, capable of recognizing and erasing gliomas [226]. Surface modification of EVs with targeted peptides and nanobodies can enhance the delivery of therapeutic molecules to cancer cells with corresponding ligands, thereby having endogenous therapeutic properties and reducing side effects [227]. Click chemistry and hydrophobic insertion are common methods for chemically modifying EVs. The approach of click chemistry occurs through the reaction between an azide or alkynene group expressed on EVs and an alkynene or azide group bound to a targeted ligand [228, 229]. It is reported that EVs generated by click chemistry between the alkyne group of reactive dibenzylcyclootyne (DBCO) and azide functionalized integrin αvβ3-targeting c (RGDyK) via intravenous administration peptide showed the targeting of the ischemic brain followed by inhibiting cell apoptosis and inflammatory response [28]. Whether these surface modification methods can also be used to enhance the binding and targeting of ADEVs to ischemic brain needs to be further investigated.

#### 6.3.2 Engineering strategies for enhanced stability and bioavailability

Despite the progress made in the research of natural EVs for the treatment of brain diseases, further improvements are still needed to improve safety and bioavailability and promote their clinical translation. The first is to improve the biological activity of EVs. The selection of appropriate cell lines to generate EVs and optimization of their production methods may increase their therapeutic efficacy [230]. Secondly, specialized bioengineering of EVs to carry therapeutic cargo molecules can improve drug stability and bioavailability. The ways of loading cargo to EVs include exogenous loading and endogenous loading [231]. Exogenous loading involves directly attaching cargos to the EV surface or membrane using techniques like ultrasound, electroporation, extrusion, freeze-thaw cycles, and chemical agents. This approach helps to improve efficiency for large-scale production, but causes EVs aggregation and lysis as well as membrane structure disruption [232]. On the other hand, endogenous loading, the genetic engineering of parental cells to secrete EVs with the desired protein, does not destroy the membrane structure of EVs, but the limitation is that it lacks control over the loaded drug's quantity [233]. Additionally, surface modification or genetic engineering can enhance EVs bioavailability in specific tissues or cells. Overexpressing proteins required for EVs can also be achieved by transfection of donor cells with plasmids encoding targeted peptides and proteins. For example, EVs modified with iRGD peptide (CRGDKGPDC) with high affinity to αv integrin efficiently deliver doxorubicin (DOX) to breast cancer cells to inhibit tumor growth without toxicity [234]. Liu et al. prepared heptapeptide (Hep)-loaded macrophage-derived exosomes (EXO-Hep), which reduced mitochondrial damage in astrocytes, promoted the transfer of healthy astroglia-derived mitochondria to neurons, and attenuated mitochondria-mediated neuronal damage by inhibiting the Drp1/Fis1 interaction after ischemia-reperfusion[235].

Therefore, it is necessary to consider engineering strategies such as optimization preparation processes, surface modifications, drug encapsulations, enhanced targeting approaches, stability optimization, and biosafety considerations when engineering ADEVs. These strategies can contribute to the advancement and successful translation of ADEV-based therapies for brain diseases.

## 7. Preclinical and clinical studies on ADEVS in ischemic stroke

### 7.1 Overview of in vitro and in vivo studies investigating the therapeutic potential of ADEVs

Insights gained from in vivo ischemic animal models and in vitro experiments have shed light on the characteristics and potential roles of ADEVs in the context of ischemic stroke. After stroke, ADEVs exhibit an altered cargo composition, including increased levels of miRNA, proteins, metabolites and other cargoes. These cargoes facilitate intercellular communications and trigger essential signaling pathways. For example, ADEVs can participate in the regulation of autophagy in neuronal cells, thereby reducing apoptosis, oxidative stress, hypoxia-ischemia induced neuroinflammation and the expression of pro-inflammatory factors. In addition, ADEVs can also promote axon regeneration and plasticity. In vivo studies have also shown that ADEV reduces neurological deficits, infarct volume, brain edema, and neuronal damage. ADEVs can also be involved in regulating the polarization of microglia after stroke, promoting the proliferation and migration of oligodendrocytes, and recruiting peripheral leukocytes for tissue infiltration.

### 7.2 Clinical trials and translational research exploring ADEV-based therapies

Unfortunately, there are still few clinical trials or translational studies of ADEVs in stroke. However, emerging studies have shown that EVs are involved in the regulation of physiological and pathological processes and promoting brain remodeling after ischemic stroke by transferring cargos. Therefore, EVs are considered to be promising biomarkers for early diagnosis as well as potential therapeutic agents for ischemic stroke [236]. In addition to clinical trials, several translational studies are underway aimed at bringing ADEV-based therapies into clinical practice. These efforts encompass improving the methods for producing and purifying EVs, delving into the therapeutic mechanisms underlying their effects, and optimizing factors such as dosage and administration routes. Understanding the composition of ADEV and developing advanced methods to engineer ADEV cargo with desired miRNA and proteins will further pave the way for clinical applications of ADEVs.

### 7.3 Mechanistic insights from preclinical and clinical studies

There are only two registered clinical trials (NCT03384433) involving EVs for stroke treatment. A randomized controlled trial (RCT) was recently reported to evaluate the safety and disability index of placental MSC-EVs injected into the brain parenchyma of five malignant middle cerebral artery infarct (mMCAI) patients. The results demonstrated the safety of local injection of EVs after mMCAI, with no adverse effects in the short or long term (three months) and improvements in multiple functional indicators, although a few patients in the study did not yield reliable conclusions [237]. The expression of brain-specific miR-9 and miR-124 was significantly increased in serum exosomes of AIS patients. In addition, this increase was positively correlated with infarct volume, serum expression of the proinflammatory factor IL-6, and National Institutes of Health Stroke Scale (NIHSS) score. This suggests that serum exosomal miR-9 and miR-124 are promising biomarkers for diagnosing AIS and assessing the extent of ischemic injury [238]. Another noteworthy study is a phase I/II clinical trial evaluating the safety and efficacy of EVs derived from allogeneic adipose MSCs administered for nasal drip in patients with AD (NCT0438982) [239]. Numerous studies have shown that EVs regulate receptor cells and post-stroke processes mainly through miRNA. Here, the changes of miRNA content carried by fluid-derived EVs in patients with acute stroke, subacute stroke and transient ischemic attack are summarized in articles [240]. This collection of evidence illustrated that changes in cargos of EVs can be used as ideal biomarkers for clinical diagnosis and monitoring of ischemic stroke. As potential biomarkers, ADEVs need to be further explored in clinical trials in the future.

## 8. Safety considerations and regulatory aspects

### 8.1 Evaluation of the safety profile of ADEVs in ischemic stroke treatment

The safety evaluation of ADEVs in the treatment of ischemic stroke is still in the research stage, and no clear conclusion has been reached. In the treatment of ischemic stroke, EVs are considered to hold potential therapeutic effects and can promote the repair of brain tissue by delivering molecules required for repair and protection of neurons. However, further studies are needed to evaluate the safety of ADEVs in the treatment of stroke. The current research mainly focuses on animal models and in vitro experiments, and large-scale clinical trials yet to be conducted. further investigations are essential to comprehensively understand the potential risks, adverse effects, and safety parameters associated with ADEVs treatment. It is imperative to thoroughly assess the safety profile of ADEVs, particularly in the context of human application, before moving forward with widespread clinical implementation.

### 8.2 Regulatory considerations and approval process for ADEV-based therapies

Regulatory considerations include safety assessment, efficacy evaluation, manufacturing quality control, and ethical review [241]. First, regulatory agencies typically require safety assessments for extracellular vesicle therapies, which involve in vitro and animal studies to assess potential risks to patients. The second is to conduct clinical trials to evaluate the efficacy and effectiveness of ADEVs therapy. The third aspect is to develop strict and standardized production quality control criteria to ensure the quality and consistency of ADEVs. Lastly, when research involves human trials, ADEVs treatment should be required to undergo ethical review to ensure that the research complies with ethical principles and legal requirements.

Meanwhile, the approval process should follow these steps:

1. Application submission. Research institutions or pharmaceutical companies are required to submit regulatory applications for ADEVs therapies, including relevant safety and efficacy data.
2. Review and Evaluation. Regulatory authorities review and assess applications, including clinical trial design, manufacturing quality control, and ethical compliance.
3. Approval and Regulation. If the application is approved, the regulator will establish the corresponding regulatory provisions and conditions and regulate and supervise the manufacture and use of the therapy.

## 9 Conclusions

### 9.1 Recap of the therapeutic mechanisms and potential of ADEVs in ischemic stroke

Because ADEVs have not been tested and applied clinically, based on rodent ischemic models and in vitro experiments, it can be concluded that after ischemic stroke, the cargoes such as miRNAs, proteins, metabolites, etc. in ADEVs are altered. For example, ADEVs contain a large number of neuroprotective factors and growth factors, including FGF2, VEGF, and ApoD. Under oxidative stress conditions stimulated astrocytes secrete EVs with altered cargo profiles, including significant amounts of HSPs, PrP, and synapsin 1. Moreover, when astrocytes are exposed to proinflammatory factors such as IL-1β and TNF-α, LCN2 and other cargoes in ADEVs are released, leading to the loss and damage of neurons. By regulating neuronal autophagy and activating signaling pathways, ADEVs can reduce neuronal apoptosis, oxidative stress, neuroinflammation and the expression of pro-inflammatory factors caused by hypoxia-ischemia and promote axonal regeneration and plasticity. ADEV can also reduce the neurological deficit, infarct size and brain edema in ischemic animals. In addition, ADEV can be involved in regulating the polarization of microglia after stroke, promoting the proliferation and migration of oligodendrocytes, and recruiting peripheral leukocytes for tissue infiltration.

### 9.2 Limitation of current studies

There are several limitations of current studies. 1. The current studies largely focus on the communication between ADEVs and neurons, while ignoring how ADEVs regulate other cells in the CNS, including microglia, oligodendrocyte, endothelial cells etc. More attention should be paid to the information transfer of ADEVs to other cells. 2. In these studies, ADEVs were routinely isolated from 2D cultured mouse/rat astrocytes, which may not fully replicate the clinical scenario. ADEVs isolated from 3D cultured human astrocytes should be tested in future. 3. In vitro studies have used neuron cell lines but not primary neurons. 4. Most studies incubate neurons with ADEVs to investigate how ADEVs regulate the function of brain, which does not mimic the clinical scenario and neglects the effects of ADEVs on other brain cells. Brain organoid should be utilized to better simulate clinical situations. 5. Few studies reported batch-batch variation regarding ADEVs phenotype and function. 6. Most animal models for ischemic stroke research are young adult mice. Therefore, more preclinical studies on aged rodents and primates are needed for future clinical translation. 7. The mechanism of ADEVs effect on brain function is still unclear. Most studies focused on one miRNA or gene, however, how interaction of multiple genes synergistically regulate the function of ADEVs needs further exploration.

### 9.3 Summary of current challenges and prospects in ADEV-based therapies

However, ADEVs therapy also has some limitations, such as the need for the consistent and standardized purification of ADEVs, ensuring their quality and quantity; identification and validation of specific biomarkers associated with ADEVs; a comprehensive understanding of the diverse cargoes carried by ADEVs [242]; Determining the optimal administration route, dosage, and timing of ADEVs. Furthermore, the current studies only focus on the communication between astrocytes and neurons, and more attention should be paid to the information transfer of other cells. Besides, there are few in vivo studies on ADEVs, and most animal models for ischemic stroke research are mice. Therefore, more preclinical studies on primates are needed.

ADEVs play an important role in neural regeneration and protection after stroke. ADEVs hold potential as biomarkers in liquid biopsy, showing great prospects in the diagnosis, prognosis, and treatment of ischemic stroke. The function of ADEVs is closely related to the cargo they transport. Wang et al. summarized the differentially expressed miRNAs in control and OGD/R-treated ADEVs by smallRNA sequencing, and further expression analysis and target prediction of these differentially expressed miRNAs in ischemic stroke can be performed in the future [243, 244]. Therefore, engineered ADEVs can be an effective treatment for stroke. Currently, stem cell therapy [245, 246] and in vivo reprogramming techniques [247] are regarded as promising approaches, such as promoting neural regeneration after brain injury. In addition, organoids can be used as stand-ins for patients in vitro to accurately reflect the efficacy of drugs in vivo, and can also play a more important role in the development of new drugs [248]. However, ADEVs have not been reported to be detected in brain organoids. In the future, these treatments may offer new hope to stroke patients.

In addition, adopting a multi-dimensional perspective is also an important means for in-depth study of the brain. Traditional research methods often focus on one aspect, whereas a multi-dimensional viewpoint enables the simultaneous consideration of numerous factors, spanning molecular, cellular, and systemic levels. Advances in hardware technology and information technology also provide strong support for brain research. For example, the development of brain imaging technology allows us to non-invasively observe the activity of the brain and thus reveal its function and structure [249]. At the same time, the application of information technology can help us process and analyze a large amount of brain science data, to extract valuable information. Finally, the establishment of high-quality clinical cohorts stands as a foundational requisite for investigating brain function and pathology. By collecting large amounts of clinical data and samples, we can establish representative research cohorts to better understand the mechanisms and develop precise treatments of brain disorders.

### 9.4 Final thoughts on the clinical implications and therapeutic potential of ADEVs in ischemic stroke treatment

Overall, ADEVs therapy emerges as a promising approach to treat patients with cerebral ischemia, supported by validation through animal models and in vitro experiments. ADEVs-mediated therapy is superior to cell therapy in terms of scalable production and safety. However, continuous efforts are needed to control the heterogeneity of cargoes carried, optimization of vesicle surface molecules, and enhance vesicle yield. At present, ADEVs treatment is still in the process of development. So far, only two clinical trials of MSC-EVs have been conducted to evaluate the potential of EVs therapy in brain diseases. Moreover, in addition to extensive quality control, further clinical studies are needed to address the limitations of EVs therapy in clinical progression. Finally, ADEVs characterization and application routes and dosages should be defined. The development of distinct engineered ADEVs tailored to different stages and characteristics of cerebral ischemia remains a challenging aspect that requires careful resolution.
